# Induction and modulation of persistent activity in a layer V PFC microcircuit model

**DOI:** 10.3389/fncir.2013.00161

**Published:** 2013-10-09

**Authors:** Athanasia Papoutsi, Kyriaki Sidiropoulou, Vassilis Cutsuridis, Panayiota Poirazi

**Affiliations:** ^1^Institute of Molecular Biology and Biotechnology, Foundation for Research and Technology-HellasHeraklion, Greece; ^2^Department of Biology, University of CreteHeraklion, Crete, Greece

**Keywords:** prefrontal cortex, computer model, intrinsic mechanisms, dADP, NMDA, GABA_B_

## Abstract

Working memory refers to the temporary storage of information and is strongly associated with the prefrontal cortex (PFC). Persistent activity of cortical neurons, namely the activity that persists beyond the stimulus presentation, is considered the cellular correlate of working memory. Although past studies suggested that this type of activity is characteristic of large scale networks, recent experimental evidence imply that small, tightly interconnected clusters of neurons in the cortex may support similar functionalities. However, very little is known about the biophysical mechanisms giving rise to persistent activity in small-sized microcircuits in the PFC. Here, we present a detailed biophysically—yet morphologically simplified—microcircuit model of layer V PFC neurons that incorporates connectivity constraints and is validated against a multitude of experimental data. We show that (a) a small-sized network can exhibit persistent activity under realistic stimulus conditions. (b) Its emergence depends strongly on the interplay of dADP, NMDA, and GABA_B_ currents. (c) Although increases in stimulus duration increase the probability of persistent activity induction, variability in the stimulus firing frequency does not consistently influence it. (d) Modulation of ionic conductances (*I*_*h*_, *I_*D*_*, *I*_sAHP_, *I*_caL_, *I*_caN_, *I*_caR_) differentially controls persistent activity properties in a location dependent manner. These findings suggest that modulation of the microcircuit's firing characteristics is achieved primarily through changes in its intrinsic mechanism makeup, supporting the hypothesis of multiple bi-stable units in the PFC. Overall, the model generates a number of experimentally testable predictions that may lead to a better understanding of the biophysical mechanisms of persistent activity induction and modulation in the PFC.

## Introduction

A number of studies have revealed the existence of small, tightly interconnected “clusters” of neurons in cortical areas (Seung, 2009; Feldt et al., 2011), whose role in cognitive processes remains a mystery. In the prefrontal cortex (PFC) in particular, a region strongly associated with complex behaviors such as working memory, attention and decision making (Curtis and Lee, 2010; Gazzaley and Nobre, 2012), understanding the function of these clusters becomes greatly important (Wang et al., 2006; Otsuka and Kawaguchi, 2008). Toward this goal, numerous studies begin to characterize the origin of these assemblies aiming to link structure with function (Cutsuridis et al., 2009). A few studies suggest that they may serve as computational modules in receptive field formation (Ko et al., 2011) and the generation of Up-and-Down states both *in vitro* (Cossart et al., 2003) and *in vivo* (Yassin et al., 2010).

In the PFC, the old notion of micro-columns with iso- and cross-directional inhibition has been hypothesized to support persistent activity (Rao et al., 1999), the cellular expression of working memory (Goldman-Rakic, 1995), although the existence of these structures remains debatable (Hill et al., 2012; Perin et al., 2013). The features of stimulus-selective PFC persistent activity have been previously investigated using attractor models of abstract or conductance-based large-scale networks of point neurons (Wang, 2001; Brody et al., 2003). These studies have greatly contributed to linking sustained activity with different types of working memory (Compte, 2006), have uncovered the importance of the NMDA and other regenerative currents in the stability of persistent activity (Wang, 1999; Tegnér et al., 2002) and have identified the significance of excitation-inhibition balance in maintaining persistent states (Fellous and Sejnowski, 2003). Although these models are able to reproduce the *in vivo* characteristics of persistent activity, such as stimulus coding, they, in great part, neglect the differential expression of synaptic and ionic mechanisms along the somato-dendritic compartments of neurons that allows for functional compartmentalization and enables them to perform difficult computations relevant to their neuronal function (Segev and London, 2000; Papoutsi et al., 2013). In addition, the underlying anatomical micro-architecture is considered to participate in the observed electrophysiological output and the computations performed in each brain region, and thus models should also be constrained by realistic anatomical data.

On the other hand, the layer-specific PFC microcircuits of few neurons described in this study, are characterized by highly reciprocal connections and facilitating synapses (Wang et al., 2006) and have been proposed to support the spontaneous emergence of Up and Down states (Shu et al., 2003), a phenomenon linked to persistent activity (Seamans, 2003). It remains unknown, however, whether such isolated microcircuits can indeed support persistent firing, how their properties may be influenced by changes in their intrinsic and/or synaptic mechanisms and whether they can express stimulus-specific coding, such as the one seen in large scale networks (Romo et al., 1999).

To address these questions, we developed a computational microcircuit model of layer V PFC neurons. The network model, its neuronal components, their connectivity, their synaptic and firing properties were validated against numerous experimental data (Van der Loos and Glaser, 1972; Kawaguchi and Kubota, 1993; Buhl et al., 1997; Markram, 1997; Markram et al., 1997; Tamás et al., 1997a,b; Angulo et al., 1999; Dombrowski et al., 2001; Bacci et al., 2003; Nasif et al., 2005; Durstewitz and Gabriel, 2007; Thomson and Lamy, 2007; Woo et al., 2007; Wang et al., 2008; Sidiropoulou et al., 2009). We used the model to induce persistent activity and characterize the conditions that allow its emergence. We then focused on the mechanisms (synaptic and intrinsic) that could modulate persistent firing, providing new insights on how PFC microcircuits may serve as key players in working memory formation. Our simulations show that: (1) the microcircuit supports persistent activity only in cases of enhanced NMDA currents, in the absence of intrinsic regenerative currents, (2) the properties of the stimulus do not seem to be coded in the probability of persistent activity induction, (3) the *I*_sAHP_ and D-type K currents reduce the probability for persistent activity induction while the h-current and the dendritic R-type calcium currents enhance emergence of persistent activity states, and (4) changes in the dADP, NMDA, and GABA_B_ currents strongly affect the temporal properties of persistent activity with dADP facilitating its induction.

## Materials and methods

### Pyramidal neuron model

The layer V PFC pyramidal neuron model was adapted from (Durstewitz et al., 2000; Durstewitz and Gabriel, 2007) and validated against experimental data. A simplified morphology was used in order to dissect the role of intrinsic and synaptic currents from that of a detailed dendritic tree. It consisted of five compartments: soma, axon, basal dendrite, proximal apical dendrite, and distal apical dendrite. The somatic, proximal and distal apical dendritic compartments included Hodgkin–Huxley-type transient (*I*_Naf_) and persistent (*I*_Nap_) Na^+^ currents, voltage-dependent K^+^ currents (*I*_Kdr_; *I_A_*; *I_D_*), a fast Ca^++^ and voltage-dependent K^+^ current, (*I*_fAHP_), a slow Ca^++^-dependent K^+^ current (*I*_sAHP_), a hyperpolarization-activated non-specific cation current (*I*_*h*_), a low-voltage activated calcium current (*I*_caT_) and four types of Ca^++^- and voltage-dependent calcium currents (*I*_caN_; *I*_caR_; *I*_caL_; *I*_CaT_). The basal dendrite included a sodium (Na^+^) current, a delayed K^+^ rectifier current, a persistent Na^+^ current, an A-type K^+^ current, a D-type K^+^ current, an N-type Ca^2+^ current and an h current. The axon included a sodium (Na^+^) current and a delayed rectifier K^+^ current.

**Figure 1:**
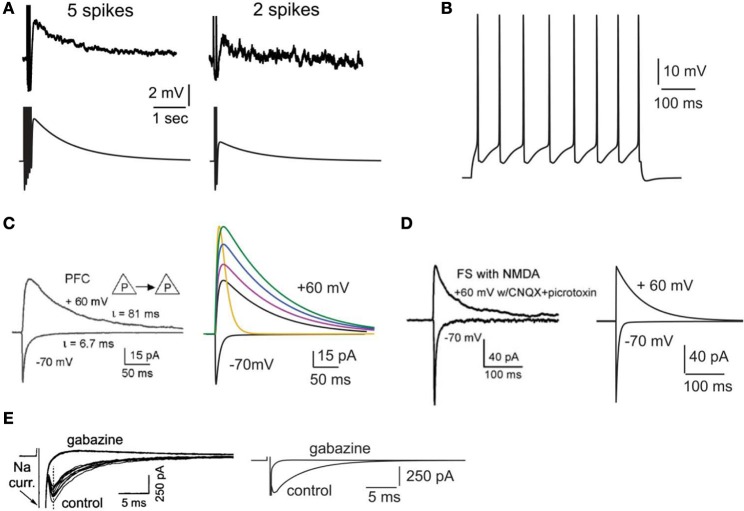
**Validation of intrinsic and synaptic properties of pyramidal and interneuron models. (A)**. Validation of the delayed afterdepolarization (dADP). Top traces: experimental recording of a layer V PFC pyramidal neuron following 5 or 2 actions potentials in response to current clamp stimulation at 20 Hz (reprinted with permission from Sidiropoulou et al., 2009). Bottom traces: simulated dADP in a single pyramidal model neuron following 5 or 2 actions potentials in response to current clamp stimulation at 20 Hz and activation of the dADP mechanism. The action potentials are truncated for better visualization of the dADP. **(B)** Voltage response of the pyramidal neuron model to a 0.25 nA, 500 ms step pulse. **(C)** Validation of the kinetics and iNMDA-to-iAMPA ratio at the basal dendrites of pyramidal neuron models. Left: experimental trace (reprinted with permission from Wang et al., 2008), showing the response in the soma of a layer V prefrontal pyramidal neuron after stimulation of a pyramidal–pyramidal pair under voltage clamp conditions at −70 mV (iAMPA) and at +60 mV under blockage of AMPA receptors (iNMDA). Copyright (2008) National Academy of Sciences, U.S.A. Right, black traces: model reproduction of iAMPA (−70 mV) and iNMDA (+60 mV) under the same protocol, using the number of synapses each pyramidal sends to the other (5 synapses). Purple trace corresponds to iNMDA-to-iAMPA ratio = 1.5, blue to iNMDA-to-iAMPA ratio = 1.9, green to iNMDA-to-iAMPA ratio = 2.3. Yellow trace: Voltage clamp trace of the NMDA current for iNMDA-to-iAMPA ratio = 2.3, where the decay time constant τ_NMDA_ is reduced from 107 ms to 18 ms. **(D)** Validation of the kinetics and iNMDA-to-iAMPA ratio of the interneuron model. Left: experimental trace (reprinted with permission from Wang et al., 2008), showing the response in the soma of a fast spiking interneuron after stimulation of a pyramidal neuron under voltage clamp conditions at −70 mV (iAMPA) and at +60 mV (iNMDA). Right: model reproduction of AMPA (−70 mV) and NMDA (+60 mV) currents under the same protocol using the same number of synapses a pyramidal neuron sends to the interneuron (2 synapses). **(E)** Validation of the autaptic inhibitory current. Left: experimental trace of autaptic inhibitory current in response to voltage clump steps from −70 to +10 for 1 ms (reprinted with permission from Bacci et al., 2003). The current disappears after application of the GABAA blocker gabazine. Right: Reproduction of the results using the same protocol in the model.

The calcium-activated non-selective cation (CAN) current (Sidiropoulou and Poirazi, 2012) that results in the generation of the delayed after depolarization (dADP) was activated in specific cases. The kinetics of the dADP mechanism were fit to the experimental recordings of (Sidiropoulou et al., 2009) as shown in Figure 1A. Parameters of the dADP mechanism were adjusted so that the dADP was activated following more than 4 spikes and had decay kinetics in the order of a few (~3) s (Figure 1A). In control conditions, unless otherwise noted, the dADP was deactivated (0 mV). When activated, its amplitude was within the experimentally reported range (1–6 mV) as per (Sidiropoulou et al., 2009).

Active properties of the neuron model were validated according to the experimental results of (Nasif et al., 2005). Specifically, input resistance *R_IN_* was set to 80 MΩ, the current needed to generate an action potential was 0.23 nA and the action potential threshold was −43.5 mV. The pyramidal neuron responded to a depolarizing current pulse (0.25 nA, 500 ms) with eight spikes, as shown in Figure 1B.

The dimensions of the somatic, axonic, and dendritic compartments of the pyramidal model cell are presented in Table 1. The passive parameters of the model neuron are listed in Table 2, while the active ionic properties are listed in Table 3.

**Table 1:** **Structure of model cells**.

	**Length (μm)**	**Diameter(μm)**
**PYRAMIDAL CELL**		
Soma	75	10.14
Basal dendrite	150	1
Proximal apical dendrite	400	3.4
Distal apical dendrite	400	2.6
Axon	113.22	1.1
**INHIBITORY INTERNEURON**		
Soma	53	42
Axon	113.22	0.7

**Table 2:** **Passive properties of all compartments of pyramidal cells and inhibitory interneurons in the microcircuit**.

	***C_M_*, μF/cm^2^**	***R_A_*, ohm/cm**	***R_M_*, kΩ cm^2^**
**PYRAMIDAL CELL**			
Soma	1.2	100	16
Basal dendrite	2	100	5.9
Proximal apical dendrite	1.2	150	5.9
Distal apical dendrite	1.2	150	5.9
Axon	1.2	150	12
**INHIBITORY INTERNEURON**			
Soma	1.2	150	15
Axon	1.2	150	15

**Table 3:** **Active ionic properties of pyramidal neurons**.

**Mechanisms**	**Soma**	**Axon**	**Basal dendrites**	**Proximal apical dendrites**	**Distal apical dendrites**
Sodium conductance, S/cm^2^	0.1809	0.18	0.0018	0.005	0.0036
Delayed rectifier K^+^, S/cm^2^	0.0216	0.0054	0.0054	2.16e-5	5.4e-6
Persistent sodium, S/cm^2^	0.18e-5	0	1.8e-5	5.4e-5	1.8e-4
A-type K^+^, S/cm^2^	7e-4	0	7e-4	7e-5	7e-5
D-type K^+^, S/cm^2^	1.026e-3	0	6e-4	1.2e-3	1.2e-3
N-type calcium, S/cm^2^	2e-5	0	6e-5	6e-5	1e-3
T-type calcium, S/cm^2^	6e-6	0	0	6e-5	6e-6
CaR, S/cm^2^	3e-5	0	0	9e-6	1.5e-3
L-type calcium, S/cm^2^	3e-5	0	0	1.9e-4	3.6e-6
sAHP, S/cm^2^	1.4e-1	0	0	2.75e-3	2.75e-5
fAHP, S/cm^2^	2.2e-3	0	0	2.2e-5	2.2e-6
H-current, S/cm^2^	7.2e-6	0	9e-6	1.4e-5	9e-5
dADP current, S/cm^2^	0	0	0	0	0
Calcium diffusion model	Yes	No	Yes	Yes	Yes
*E_na_*, mV	+55	+55	+55	+55	+55
*E_K_*, mV	−80	−80	−80	−80	−80
*E_ca_*, mV	+140	–	+140	+140	+140
*E_h_*, mV	−10	–	−10	−10	−10
*E_L_*, mV	−65	−65	−65	−65	−65

### Interneuron model

The interneuron model (fast spiking, FS) was adapted from (Durstewitz and Gabriel, 2007) (Model-DB, accession number 82784), with the addition of an axonal compartment and validated against experimental data. The somatic compartment included a Na^+^ current (*I*_Naf_) and two types of K^+^ currents (*I*_Kdr_; *I_D_*). The axon included a Na^+^ current (*I*_Naf_) and a delayed rectifier K^+^ currents (*I*_kdr_). The resting membrane potential (*V_m_*) was set to −70 mV and the input resistance *R_IN_* to 207 MΩ (Kawaguchi and Kubota, 1993; Cauli et al., 1997). The interneuron responded to step pulses of 0.2 nA and 0.35 nA with a firing frequency of 60 and 90 Hz, respectively, in agreement with (Kawaguchi and Kubota, 1993; Cauli et al., 1997; Wang and Gao, 2009).

The dimensions of the somatic and axonic compartments are presented in Table 1. Passive and active ionic properties of the interneuron model are listed in Tables 2 and 4, respectively.

**Table 4:** **Active ionic properties of inhibitory interneurons**.

**Mechanisms**	**Soma**	**Axon**
Sodium conductance, S/cm^2^	0.225	0.54
Delayed rectifier, S/cm^2^	0.018	0.018
D-type K^+^, S/cm^2^	7.25e-5	0
*E_na_*, mV	+55	+55
*E_K_*, mV	−80	−80
*E_L_*, mV	−70	−70

### Synaptic properties

The AMPA current in the pyramidal cell was tuned to match the experimental data of (Wang et al., 2008): under voltage clamp conditions at −70 mV and activation of 5 synapses (simulating the monosynaptic connections between layer 5 pyramidal neurons), the amplitude and kinetics of the AMPA current fit the experimentally reported values as shown in Figure 1C.

The amplitude and kinetics of the NMDA current in the pyramidal cell were also validated with voltage clamp at +60 mV under conditions of blocked AMPA, Na^+^, and K^+^ currents as per (Wang et al., 2008) and are shown in Figure 1C (black traces). The baseline value for the iNMDA-to-iAMPA ratio (measured as the ratio of the peak amplitude of the iNMDA and the iAMPA under voltage clamp at +60 mV and −70 mV, respectively), at the basal dendrite was set to 1.1 as per (Wang et al., 2008). Since the NMDA current in the PFC increases either by dopamine (Seamans et al., 2001; Wang and O'Donnell, 2001) or due to the recruitment of extrasynaptic receptors (Chalifoux and Carter, 2011), in certain experiments we varied the iNMDA-to-iAMPA ratio (iNMDA increased while iAMPA remained unaltered) to values of 1.5, 1.9, and 2.3 (i.e., 36, 72 and 100% increase). All ratios were calculated under voltage clamp conditions and the respective NMDA traces are shown in Figure 1C (purple, blue, and green traces). For another set of experiments we decreased the decay time constant of the NMDA current from τ = 93 ms to τ = 18 ms (Figure 1C, yellow trace). For the proximal apical dendrite, the iNMDA-to-iAMPA ratio was half of the baseline basal dendrite value, according to (Dodt et al., 1998).

The AMPA and NMDA EPSC kinetics in the interneuron model were validated against data from fast spiking layer V PFC interneurons, by holding the membrane potential of the interneuron at −70 mV and +60 mV, respectively, as per (Wang and Gao, 2009). The corresponding amplitudes were also validated according to (Wang and Gao, 2009) and the results are shown in Figure 1D. Finally, for the validation of the interneuron autapses, a three-step voltage clamp was used (−70 mV to 10 mV to −70 mV) that resulted in a self-inhibitory current of ~350 pA (Figure 1E). It should be noted that only during the validation of this synaptic current, the reversal potential of Cl^−^ was adjusted from −80 to −16 mV, in order to reproduce the experimental set up of (Bacci et al., 2003). During the simulations we assumed physiological reverse potential (−80 mV).

The conductance of the GABA_A_ mechanism was set so that the amplitude of the mIPSC was 10 pA at a holding potential of −65 mV, as per (Woo et al., 2007). Finally, we implemented the slow inhibitory synaptic current (iGABA_B_). Physiological data regarding the iGABA_B_-to-iGABA_A_ ratio in layer V pyramidal neurons are conflicting. Application of GABA on layer V pyramidal neurons was shown to result in a GABA_B_ response that is 80% of the amplitude of the GABA_A_ response (Eder et al., 2001), while dual-recordings between an interneuron and a pyramidal neuron resulted in pure GABA_A_ or GABA_B_ responses, with the GABA_B_ responses being elicited in about 20% of the cases (Thomson and Destexhe, 1999). Due to the discrepancy of these findings, the iGABA_B_-to-iGABA_A_ ratio was varied from 0.2 to 0.8. This ratio was measured as the peak amplitude of the GABA_B_ current over the peak amplitude of the GABA_A_ current after stimulation that resulted in the saturation of the GABA_B_ current (40 events at 100 Hz), according to (Thomson et al., 1996).

The synaptic waveform parameters and conductances of AMPA, NMDA, GABA_A_, and GABA_B_ currents are listed in Table 5.

**Table 5:** **Synaptic parameters**.

	**AMPA**	**NMDA**	**GABA_A_**	**GABA_B_**
**PYRAMIDAL CELL**				
Conductance,	0.00019 (basal)	0.25 (basal)	6.9e-4	1.05e-4
nS	0.00024 (apical)	0.22 (apical)		
Reversal potential, mV	0	0	−80	−80
Rise time, ms	0.6^*^	4.3	1.5	9.8
Fall time, ms	4.3^*^	93	14	72
**INHIBITORY INTENEURON**				
Conductance, S/cm^2^	7.5e-4	3.2e-4	5.1e-4	–
Reversal potential, mV	0	0	−80	–
Rise time, ms	0.3^*^	0.5^*^	3	–
Fall time, ms	5.5^*^	66.3^*^	24	–

* *Data adapted from voltage-clamp recordings*.

### Network

**Figure 2:**
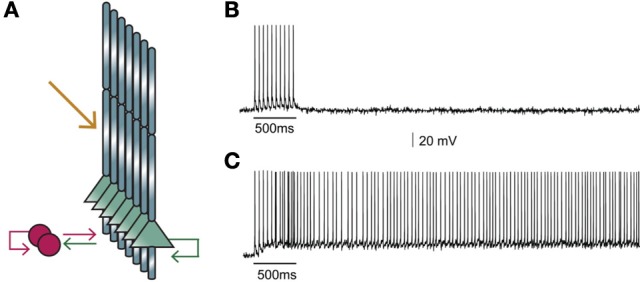
**(A)** Structure of the microcircuit that includes seven pyramidal and two interneuron models. Green arrows: excitatory connections, red arrows: inhibitory connections. Sustained activity is induced following external stimulation of the proximal apical dendrites (orange arrow). **(B)** Activation of 90 excitatory synapses impinging on the apical dendrite of each pyramidal neuron model with 10 synchronous events at 20 Hz when iNMDA-to-iAMPA ratio is 1.1 and dADP is 0 mV does not induce persistent firing. **(C)** Similar activation conditions as in **(B)**, but with iNMDA-to-iAMPA ratio = 2.3 and dADP = 0 mV, lead to persistent activity. Persistent activity is an all-or-none phenomenon: if induced, it lasts even in the absence of stimulation for the whole duration of the simulation, namely 5 s.

The network model (graphically illustrated in Figure 2A), consists of seven pyramidal neurons and two interneurons, as inhibitory neurons in the PFC constitute 25–35% of the neuronal population (Dombrowski et al., 2001). Connectivity properties including the location and number of synaptic contacts, the latencies between pairs of neurons, as well as the electrophysiological properties of their synaptic connections, were based on anatomical and electrophysiological data (see Table 6 for parameter values). The complete mathematical formalism of the model can be found in the Supporting Online Material (SOM).

**Table 6:** **Summary of synaptic connections in the microcircuit**.

**Type of connection**	**Location**	**No. of synapses**	**References**
Thalamocortical (incoming)	Proximal dendrite	90	Kuroda et al., 1998
Pyramidal recurrent	Basal dendrite	5	Markram et al., 1997
Pyramidal-to-interneuron	Soma	2	Buhl et al., 1997
Autapses in pyramidal neurons	Basal dendrite	1	Lübke et al., 1996; Tamás et al., 1997b
Interneuron-to-pyramidal	Soma	4	Tamás et al., 1997a
Autapses in the interneuron	Soma	12 synaptic contacts producing ~350pA	Tamás et al., 1997b; Bacci et al., 2003

Specifically, the microcircuit incorporates the high reciprocity of connections reported in (Wang et al., 2006). Pyramidal model neurons are interconnected at the basal dendrite through 5 synapses as reported in (Markram, 1997; Markram et al., 1997), with latencies drawn from a Gaussian distribution with μ = 1.7 ms and σ = 0.9 according to (Thomson and Lamy, 2007). Autapses that have been observed between axon arbors and basal dendrites of pyramidal cells in the neocortex (Van der Loos and Glaser, 1972), equal one third of the contacts formed between two synaptically coupled pyramidal neurons, with a mean value of 2.3 ± 0.9 according to (Lübke et al., 1996). Based on the above, we used 1 autapse per neuron. In each simulation, the precise location of the connecting synapses varied randomly across the basal dendrite of each neuron. The reasoning behind this manipulation, along with all other sources of noise (membrane fluctuations, conductance delay variability, etc.), was to represent the existence of multiple microcircuits in the PFC whose properties are not identical.

Pyramidal neurons also connect to interneurons by 1–2 synaptic contacts according to (Buhl et al., 1997). The latencies of these connections are drawn from a Gaussian distribution with μ = 0.6 ms and σ = 0.2 as per (Angulo et al., 1999). Inhibitory neurons connect to pyramidal neurons and the number of these contacts is such as to produce a unitary IPSP of amplitude ~1 mV, according to (Tamás et al., 1997a). The latencies of these connections are drawn from a Gaussian distribution with ì = 1.8 ms and σ = 0.8 as per (Thomson and Lamy, 2007).

Autaptic innervation in fast-spiking interneurons is accomplished through 12 ± 7 synaptic contacts as reported in (Tamás et al., 1997b). According to a functional study of autaptic inhibitory currents in fast-spiking neurons, a single action potential evokes an autaptic inhibitory current with peak amplitude ~350 pA and mean latency of 1.76 ± 0.07 ms (Bacci et al., 2003). Since autapses strongly regulate the spike-timing of the interneurons (Bacci and Huguenard, 2006), an autaptic inhibitory current was implemented using the protocol reported in Bacci et al. (2003).

### Stimuli

Persistent activity in the microcircuit was induced by providing external synaptic stimulation (10 pulses at 20 Hz, 90 excitatory synapses) to the proximal dendrites (yellow arrow in Figure 2A) of each pyramidal model neuron (Kuroda et al., 1998). Since neurons within a microcircuit share similar stimulus properties (Yoshimura et al., 2005; Petreanu et al., 2009), the same initial stimulus was delivered to all pyramidal neurons.

### Background noise

In order to simulate as closely as possible the noise fluctuations in the membrane potential of both pyramidal cells and interneurons that are seen *in vitro*, an artificial current with Poisson characteristics was injected in all neuronal models.

### Analysis of inter-spike interval (ISI) distributions

For the analysis of spike trains, we used single-neuron ISIs and Synchronization index. Single neuron distribution of Inter-Spike-Intervals (ISIs) was estimated for each pyramidal neuron under each condition used. In addition, in order to evaluate the synchronization or de-synchronization of pyramidal neurons during persistent activity, we also estimated the Synchronization index. For this measurement we obtained the spike trains simultaneously from the pyramidal neuron population and then we calculated the time intervals between successive spikes occurring in any of the participating pyramidal neurons. If there are no phase lags between the spike trains (neurons fire synchronously) the synchronization index will have values of zero. In general, small values of synchronization index indicate synchronicity, whereas large values indicate asynchronous spiking activity (for details of the method see (Kreuz et al., 2011). ISI distributions were compared using either the pair-wise Mann-Whitney or the Kruskal-Wallis non-parametric statistical test. Comparisons with *p*-values < 0.05 were deemed statistically significant.

### Implementation

The microcircuit model was implemented in the NEURON simulation environment (Hines and Carnevale, 2001) and simulations were executed on a parallel cluster (8 core xeon processors). Data analysis was performed using MATLAB. The source code of the PFC microcircuit model is available upon request to the corresponding author at poirazi@imbb.forth.gr. Upon publication, the model will also be available via the ModelDB database.

## Results

### Induction of persistent activity

Our first goal was to test whether the microcircuit model can generate persistent activity in response to a realistic stimulus. We found that under conditions of control NMDA currents (iNMDA-to-iAMPA ratio = 1.1) and dADP = 0 mV, activation of 90 excitatory synapses impinging on the apical dendrite of each pyramidal neuron model, (with 10 synchronous events at 20 Hz), did not lead to persistent firing (see Figure 2B). When we increased the iNMDA-to-iAMPA ratio to 2.3 and kept dADP deactivated, persistent activity emerged with a probability of 86% (see Figure 2C). Persistent activity in the microcircuit model was an all-or-none phenomenon: if induced, it lasted for the whole duration of the simulation, namely 5 s.

### Stimulus effects on persistent activity

The firing frequency, duration and stimulus-specificity of persistent activity during working memory tasks are known to be modulated by dopamine as well as other neurotransmitters (Arnsten et al., 2012), although the exact mechanisms and effects of this modulation remain unclear. In the following paragraphs, we investigate the stimulus-specific factors that can influence the firing characteristics of persistent activity in the microcircuit model.

**Figure 3:**
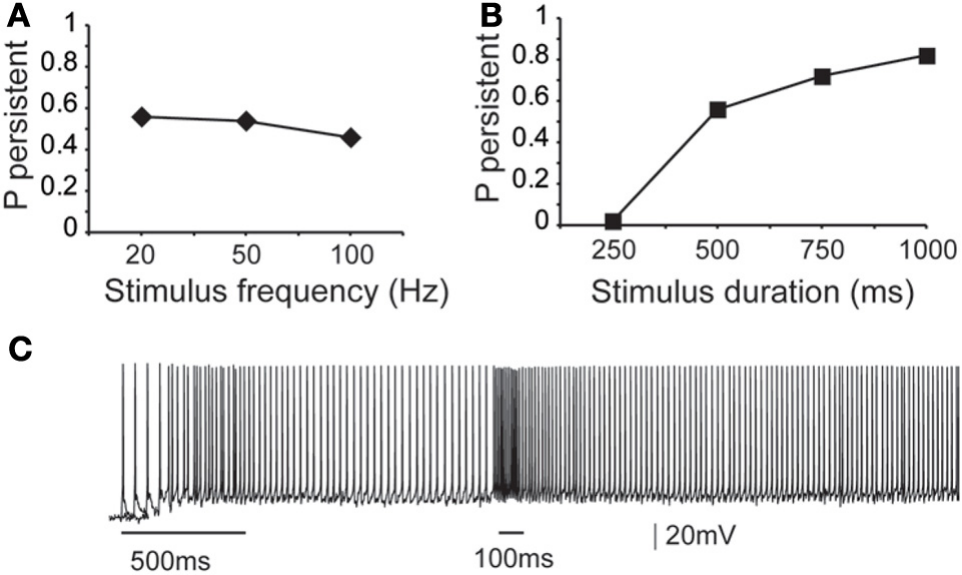
**Dependence of microcircuit spiking activity on stimulus-specific parameters. (A)** Graph showing the effect of stimulus frequency (20, 50, 100 Hz) on the probability of persistent activity initiation. Parameters used: iNMDA-to-iAMPA ratio = 2.3, iGABA_B_-to-iGABA_A_ ratio = 0.4, dADP deactivated. **(B)** Graph showing the effect of stimulus Duration (250 ms, 500 ms, 750 ms, 1 s) on the probability of persistent activity initiation. Parameters used: iNMDA-to-iAMPA ratio = 2.3, iGABA_B_-to-iGABA_A_ ratio = 0.4, dADP deactivated. **(C)** Indicative voltage trace showing the effect of a second excitatory stimulus (100 Hz, 10 events) to the pyramidal neurons. Bars indicate stimulus presentation times.

First, we asked whether the properties (firing frequency and duration) of the inducing stimulus influence the emergence and/or firing characteristics of persistent activity, using a range of input frequencies (20, 50, and 100 Hz, 10 synchronous events) and durations (0.25, 0.5, 0.75, and 1 s, input frequency 20 Hz). We found that the input frequency had no significant effect on the probability of persistent activity initiation (Figure 3A), but the duration greatly altered the microcircuit's response to the stimulus: longer stimuli were more likely to induce persistent firing (Figure 3B).

To investigate potential stimulus coding for different input frequencies and/or durations we compared the inter-spike-interval (ISI) distributions during persistent firing at a single pyramidal neuron and synchronization index in the microcircuit level. The single neuron ISI distributions were significantly different (Kruskal-Wallis test) between stimuli lasting 0.5, 0.75, and 1 s (*p* < 0.0001), with median ISI values of 11.5, 12.9, and 13.2 ms, respectively (Supplement Figures 1A–C in Supporting Online Material). Although comparison of the synchronization index of the microcircuit was significantly different (*p* < 0.001), the pair-wise comparison (Mann–Whitney *U*-test) for stimuli lasting 0.75 and 1 s were not statistically different. These results suggest that individual neurons in the microcircuit may code for differences in the stimulus duration via the use of a temporal code, but when looking at the population output of the microcircuit, this coding is weakened.

Similar results were obtained when altering the firing frequency of the input: the ISI distributions were significantly different for 20 vs. 50 or 20 vs. 100 Hz inputs (*p* < 0.0001 and *p* < 0.0001, respectively), but not for 50 vs. 100 Hz-inputs (Supplement Figures 1D–F in Supporting Online Material). Median ISI values were 11.5 ms for 20 Hz, 11.4 for 50 Hz and 11.3 ms for 100 Hz. No significant difference was found between the synchronization indexes of the microcircuit in any condition. The same analysis was performed with the dADP mechanism activated (at 2 mV), as it was previously suggested to contribute to stimulus coding at the single neuron level (Egorov et al., 2002). The results were qualitatively the same (Supplement Figure 2 in Supporting Online Material). Taken together, these data suggest that neither individual neurons nor the microcircuit as a whole can code for differences in the firing frequency of the input beyond 50 Hz. Overall, we do not find strong evidence for coding the firing characteristics of the input in our model microcircuit.

Stimulus coding may also be realized via the expression of graded persistent activity, whereby subsequent presentation of the same (or different) stimulus increases the firing frequency of persistent activity (Egorov et al., 2002). To test the microcircuit's ability to generate graded firing, we performed another set of experiments whereby a second excitatory stimulus (90 synapses activated with 10 events at 50 Hz or 100 Hz) was delivered 1 s after the initial stimulus to the proximal dendrites of all pyramidal neurons as shown in Figure 3C. Again, no differences were seen in the ISI distributions during persistent activity. These results suggest that fine tuned stimulus coding may be realized either in higher level networks or through manipulations of the intrinsic makeup of neurons by neuromodulators. We thus investigate how modulation of intrinsic neuronal properties may influence the probability of persistent activity emergence.

### Intrinsic mechanisms involved in the initiation of persistent activity

**Figure 4:**
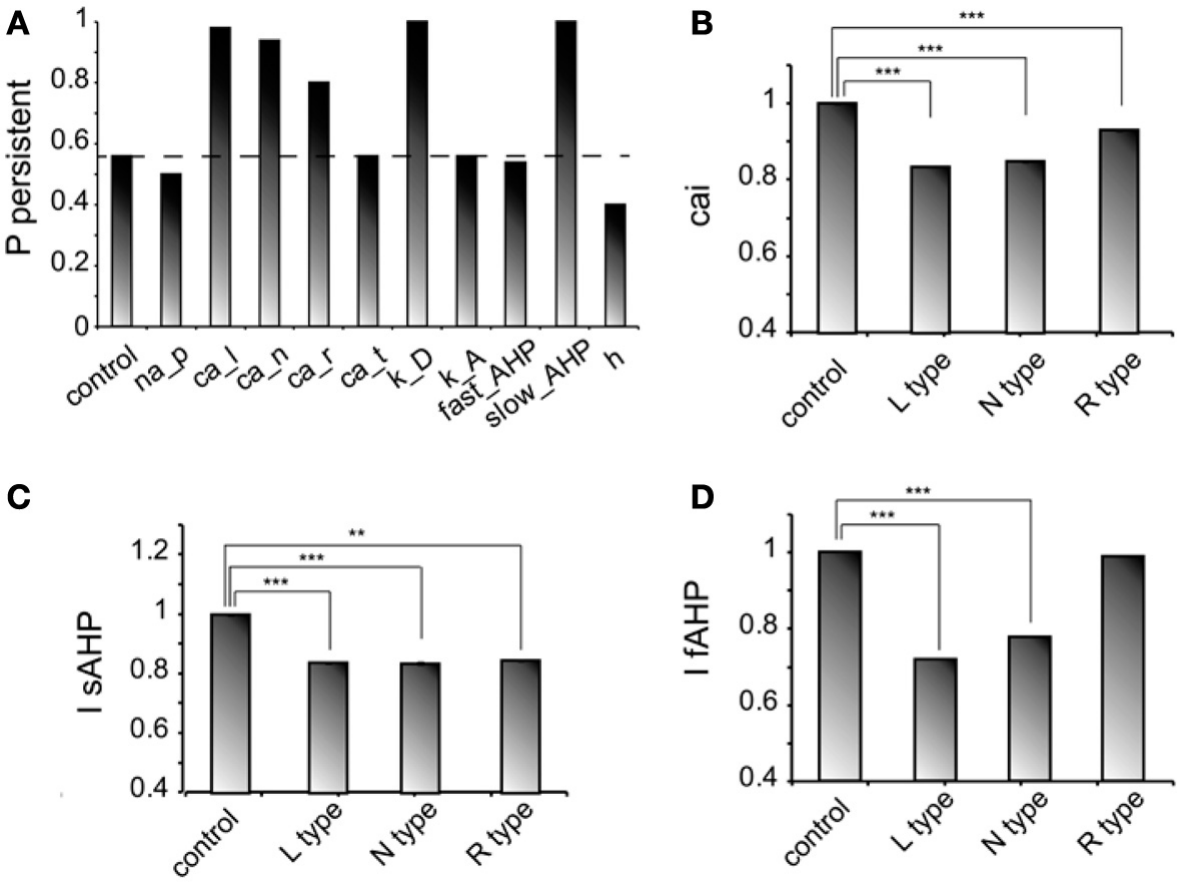
**Intrinsic conductances modulate persistent activity induction. (A)** Bar graph showing the effect on induction probability when the conductance of the various currents is reduced to 10% of its control value (iNMDA-to-iAMPA = 2.3, iGABA_B_-to-iGABA_A_ ratio = 0.4, dADP deactivated). **(B)** Bar graph showing the normalized change in intracellular calcium concentration (measured as the integral over the inducing stimulus period) with respect to the control value when L, N or R type calcium channels are blocked. **(C)** Bar graph showing the normalized change in the slow AHP current (measured as the integral over the inducing stimulus period) with respect to the control value when L, N or R type calcium channels are blocked. **(D)** Bar graph showing the normalized change in the fast AHP current (measured as the integral over the inducing stimulus period) with respect to the control value when L, N or R type calcium channels are blocked. In all cases, error bars correspond to standard error. ^**^p < 0.01; ^***^p < 0.001.

We next investigated how changes in the conductance values of membrane mechanisms, including ionic conductances, can interfere with the ability of the microcircuit to express persistent activity. We examined the contribution of various intrinsic ionic mechanisms by independently reducing their conductance to 10% of their control value and assessing the effect on persistent activity emergence. The following mechanisms were investigated: the persistent Na^+^ current, the D-type K^+^ current, the A-type K^+^ current, the h-current, the L-, N-, T- and R-type Ca^++^ currents and the currents underlying the fast and slow AHP mechanisms (*I*_sAHP_, and *I*_fAFP_, respectively). For these experiments iNMDA-to-iAMPA was 2.3, iGABA_B_-to-iGABA_A_ was 0.4 and the dADP was deactivated, leading to an induction probability of 0.54. Figure 4A shows the effect of each mechanism blockade on the probability of persistent activity emergence relative to the control.

In general, independent blockade of the D-type K^+^ current, the h-current, the L-, N- and R-type calcium current and the I_sAHP_ led to more than 15% change in the induction probability: reducing the conductance of D -type K^+^ current, the L-, N-, and R-type calcium current and the I_sAHP_ enhanced the probability of induction, whereas reducing the conductance of the *h* current reduced the probability of persistent activity emergence. Counter intuitively, blockade of the L, N, or R-type calcium currents increased the probability of persistent activity (Figure 4A). To further investigate this result, we compared the intracellular calcium accumulation (integral of the calcium concentration) and the cumulative (integral) *I*_sAHP_, and *I*_fAFP_ currents during stimulus presentation under control and calcium channel blockade conditions (Figures 4B–D). As expected, the intracellular calcium accumulation and the cumulative *I*_sAHP_ current were both significantly lower (cai: for L-type blockade *p* < 0.0001, for N-type blockade *p* < 0.0001 and for R-type blockade *p* < 0.0001, *I*_sAHP_: for L-type blockade *p* < 0.0001, for N-type blockade *p* < 0.0001and for R-type blockade *p* = 0.001) in all cases, while the cumulative *I*_fAHP_ was smaller when blocking the L, N but not the R type calcium channels (for L-type blockade *p* < 0.0001, for N-type blockade *p* < 0.0001 and for R-type blockade *p* = 0.15). Therefore, blockade of calcium channels enhances persistent activity induction via the secondary reduction of the slow after hyperpolarization current.

**Figure 5:**
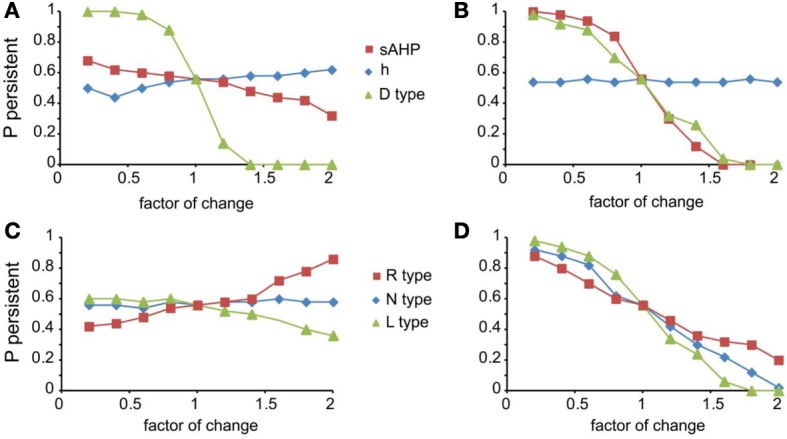
**Modulation of persistent activity by intrinsic conductances is location-specific. (A)**. Diagram showing the probability of induction when multiplying the dendritic conductance of D type, h channels and the channels that mediate the sAHP, by a factor of 0.2–2 with a step of 0.2. **(B)** Same as in **(A)** when changing the somatic conductances for these channels. **(C)** Diagram showing the probability of induction when multiplying the dendritic conductance of R, L, and N type calcium channels, by a factor of 0.2–2 with a step of 0.2. **(D)** Same as in **(C)** when changing the somatic conductances for these channels.

For the mechanisms that affected persistent activity induction, we conducted a second set of experiments aiming to dissect the effects of dendritic vs. somatic manipulations by altering their conductance values (multiplying with a factor of 0.2–2, with a step of 0.2) separately at the dendrites and the soma (Figure 5). We found that the *h*-current had a small effect on persistent activity only when conductance manipulations were done in the dendrites (Figure 5A diamonds), with the probability being proportional to the conductance. For *I*_sAHP_ and the D-type K^+^ currents (*I_D_*) somatic conductances had a prominent, inversely proportional effect on the induction probability (Figure 5B squares and triangles). For *I*_sAHP_, this effect was attenuated when manipulations took place in the dendrites (Figure 5A red squares) while the opposite was true for *I_D_* (Figure 5A green triangles). Since both currents are potassium currents with slowly inactivating characteristics that contribute to prolonged membrane hyperpolarization, the different contributions seen for dendritic manipulations can be explained by the different distribution patterns of these channels: fewer sAHP channels are present in the dendrites whereas D-type channels increase in these regions, compared to their cell body values (Korngreen and Sakmann, 2000).

Similar to the *I*_sAHP_, somatic VGCCs have a more prominent effect of the induction probability than the respective dendritic currents (Figures 5C,D). Again, induction probability is inversely proportional to the conductance values of these channels, presumably via the secondary activation of sAHP (see Figure 4C). Changes in dendritic conductances of VGCC have more subtle effects. The N-type calcium current does not affect the induction probability, whereas the effect of L-type calcium current is greatly attenuated but with the same trend as the respective somatic conductance. The R-type calcium current, however, has opposite effects, depending on its somatic or dendritic localization: dendritic modifications are proportional while somatic modifications are inversely proportional to the induction probability. The possible explanation for this observation is that R-type calcium channels contribute to bursting when located in the dendrites (Takahashi and Magee, 2009; Pissadaki et al., 2010) and to AHP when located at the soma. Thus, increasing R-type channel currents in the dendrites enhances local depolarization and perhaps contributes to dendritic spike generation, whereas increasing it in the soma does not contribute to dendritic events (Figures 5C,D).

Most importantly, our model predicts that location dependent manipulations of VGCCs and the sAHP conductances differentially influence signal integration and persistent firing.

### Excitation-inhibition balance and dADP effects on persistent activity induction

**Figure 6:**
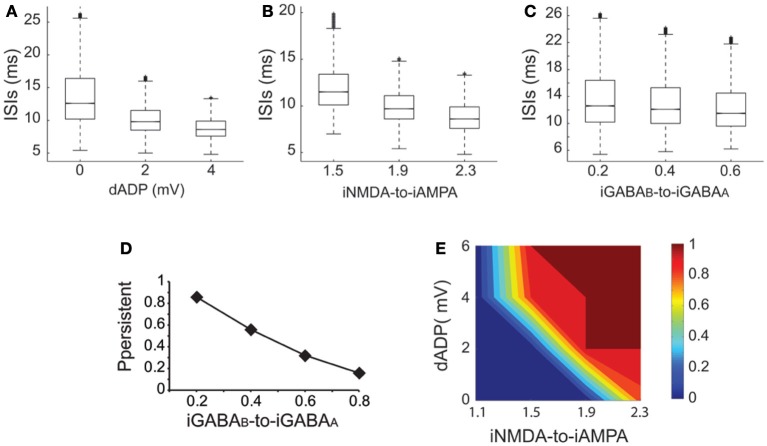
**Synaptic conductances gate and modulate the persistent excitable states. (A)** Box plot of ISI distributions recorded in a single pyramidal neuron for dADP values 0, 2, and 4 mV. **(B)** Box plot of ISI distributions recorded in a single pyramidal neuron for iNMDA-to-iAMPA ratio 1.5, 1.9, 2.3. **(C)** Box plot of ISI distributions recorded in a single pyramidal neuron for iGABA_B_-to-iGABA_A_ values 0.2, 0.4, 0.6. **(D)** Probability of sustained activity emergence in the microcircuit as a function of the iGABA_B_-to-iGABA_A_ ratio (iNMDA-to-iAMPA = 2.3, dADP = 0 mV). **(E)** 2-D contour plot showing the combined effects of the dADP mechanism (0–6 mV) and the iNMDA-to-iAMPA ratio (1.1–2.3) on the probability of persistent activity induction. Blue: failure of induction, red: induction in 100% of the trials (iGABA_B_-to-iGABA_A_ ratio = 0.4).

Working memory has been associated with the release of various neuromodulators that influence the balance of excitation-inhibition and the magnitude of the dADP (Arnsten et al., 2012) in the PFC. Therefore, we asked whether changes in these mechanisms may influence persistent activity properties. We found that increasing the amplitude of the dADP (0, 2 and 4 mV) (Figure 6A), the iNMDA-to-iAMPA ratio (1.5, 1.9, 2.3) (Figure 6B) or the iGABA_B_-to-iGABA_A_ ratio (0.2, 0.4, 0.6) (Figure 6C) is associated with smaller average ISIs at the single neuron level (and thus larger average firing frequencies), although the effect in the median values for the different iGABA_B_-to-iGABA_A_ ratios is small (*p* < 0.0001 for all cases, Kruskal–Wallis test). These findings suggest that the release of a neuromodulator that influences any of the above mentioned mechanisms will significantly change both the average and the temporal firing characteristics of the microcircuit.

To further elucidate the effect of inhibition on persistent activity, we investigated whether slow inhibitory synaptic currents mediated by the GABA_B_ receptors can counteract the permissive effects of the NMDA current. We found that large iGABA_B_-to-iGABA_A_ ratios (when iNMDA-to-iAMPA ratio = 2.3) reduced, but did not eliminate the probability of persistent activity initiation (Figure 6D), indicating that the slow excitation may not be affected severely by inhibition. Nevertheless, activation of the GABA_B_ current modulated persistent activity initiation and adds a novel parameter to the so far known biophysical mechanisms underlying persistent activity.

In addition to synaptic, intrinsic mechanisms and particularly the dADP have been linked to persistent firing at the single neuron level (Sidiropoulou et al., 2009; Sidiropoulou and Poirazi, 2012). As depicted in Figure 6E, incorporation of the dADP mechanism in a physiologically relevant range (2–6 mV) in the microcircuit model allows the emergence of persistent activity for smaller iNMDA-to-iAMPA ratios but not for a ratio of 1.1. These findings suggest that high NMDA current is a prerequisite for persistent activity initiation in the microcircuit when the dADP current is inactive, but this necessity weakens in the presence of dADP.

## Discussion

### General issues

A detailed biophysical model of a layer V PFC microcircuit has been presented. The model is extensively constrained by experimental data (Van der Loos and Glaser, 1972; Kawaguchi and Kubota, 1993; Buhl et al., 1997; Markram, 1997; Markram et al., 1997; Tamás et al., 1997a,b; Angulo et al., 1999; Dombrowski et al., 2001; Bacci et al., 2003; Nasif et al., 2005; Durstewitz and Gabriel, 2007; Thomson and Lamy, 2007; Woo et al., 2007; Wang et al., 2008; Sidiropoulou et al., 2009) casting it as a faithful representation of a biologically realistic PFC network with morphologically simplified neurons. The model conclusively shows that the expression and modulation of persistent activity can be supported by small-sized clusters of cells (microcircuits), even though previous studies have suggested otherwise (Compte et al., 2000; Compte, 2006). Indeed, computational studies investigating network properties have become more elaborate over the years, incorporating physiological intrinsic and synaptic currents in neurons as well as their connectivity properties in order to elucidate the functional advantages of cortical microcircuits (Haeusler and Maass, 2007; Sarid et al., 2007; Litwin-Kumar and Doiron, 2012; Reimann et al., 2013). This model network supports synaptically-induced persistent activity, which is tunable by changes in several intrinsic ionic conductances, namely the CAN current that underlies the dADP, the H-current, the *I*_sAHP_ and the R-type calcium current, uncovering a new role or providing further supporting evidence for these mechanisms that can be tested experimentally. Our work suggests that these microcircuits may serve as the anatomical substrate for the expression and tunable modulation of persistent activity, thus conferring a flexible short term memory system.

### What have we learned from the model?

#### Minimal anatomical substrate of persistent activity

Most studies have suggested that intact large networks of neurons are necessary for induction and maintenance of persistent activity. This statement has been supported by the fact that persistent activity is primarily observed *in vivo*, while its generation in brain slices comes in the form of up-and-down states (McCormick, 2003). In single, isolated neurons persistent activity can only be induced in the presence of neuromodulators that activate the CAN current and generate dADP (Egorov et al., 2002; Sidiropoulou et al., 2009; Sidiropoulou and Poirazi, 2012). Here, we constructed what recent experimental data claim to be the minimal functional neuronal network in the cortex and found that this network can support persistent activity in the absence of neuromodulators that activate the CAN current, but with enhanced NMDA conductance. In agreement with our predictions, small assemblies of neurons have also been reported to support persistent spiking activity in hippocampal cultures (Lau and Bi, 2005). Therefore, it is likely that the neuronal clusters shown to exist *in vivo* (Seung, 2009; Yassin et al., 2010; Feldt et al., 2011; Ko et al., 2011) represent the minimum anatomical substrate for the emergence of stimulus-induced persistent activity. One prediction of our study is that stimulus-induced persistent activity can possibly be generated in brain slices, assuming that these network structures remain intact. Therefore, the absence of persistent activity firing in brain slices could reflect an anatomical compromise of these networks, such as cut dendrites due to the different planes that dendrites extend to in the PFC (Day et al., 2005). The anatomical (Fiala et al., 2003) or synaptic (Gundersen et al., 1995; Kuenzi et al., 2000) changes that occur after slicing can potentially affect the normal physiological mechanisms needed for persistent activity to be generated, such as dendritic non-linear events. We propose that appropriate *in vitro* experiments in organotypic cultures of the PFC alone, as has already be done with co-cultures of the PFC, the ventral tegmental area (VTA), the hippocampus or the basal forebrain (Seamans, 2003), where a high degree of connectivity is reestablished, will elucidate the necessary neural substrate for persistent activity emergence in the PFC.

#### Bistable units through balanced excitation-inhibition

Many studies have proposed balanced excitation/inhibition to promote and stabilize persistent activity (Compte, 2006) and the NMDA current as the most widely accepted source of excitation: the NMDA current is crucial for the generation of spontaneous and evoked Up-states in PFC brain slices (Shu et al., 2003; Tseng and O'Donnell, 2005) and in working memory tasks (Aultman and Moghaddam, 2001). In support, it was recently shown that intracellular application of the NMDA-channel specific blocker MK-801, *in vivo* in monkeys performing a working memory task abolishes persistent activity in the PFC (Wang et al., 2013).

On the other hand, inhibitory effects remain under-characterized. In the entorhinal cortex, GABA_A_ currents are imperative for the stabilization of Up states, while GABA_B_ currents mediate the transition to the Down state (Mann et al., 2009). A recent simulation study has also shown that the NMDA/GABA_B_ currents are the perfect couple for the emergence of bistability: NMDA promotes Up states, and the GABA_B_ current underlies the Down state (Sanders et al., 2013). Our work addresses for the first time the role of the GABA_B_ current in the generation of a strongly related phenomenon, namely persistent activity. Although this current has a slow time course, we show that it can gate the induction of persistent activity (without diminishing it in the range tested), a prediction that can be experimentally tested.

#### (No) stimulus coding

We found little evidence for stimulus coding in the microcircuit model. We showed that prolonging the duration of the input enhances the probability of induction, a result that is in agreement with findings in the entorhinal cortex (Tahvildari et al., 2007). On the other hand, varying the firing frequency of the input does not consistently affect persistent firing properties. However, changes in the dADP, NMDA, and GABA_B_ currents affect the temporal (ISI distribution) firing characteristics of persistent activity, suggesting that modulation of these mechanisms, e.g., by dopamine (Seamans and Yang, 2004; Galloway et al., 2008), may allow the selective tuning of different microcircuits, enabling the appearance of multiple, semi-independent processing modules, each with its own activity dynamics. Stimulus coding observed *in vivo* (Romo et al., 1999) could in turn result from the crosstalk of multiple microcircuits, and therefore, comprise a property of a larger neuronal network and not of a small cluster of neurons.

#### Role of intrinsic mechanisms

It is well-known that the dADP contributes to persistent firing induction and maintenance, primarily at the single neuron level (Egorov et al., 2002; Sidiropoulou et al., 2009; Sidiropoulou and Poirazi, 2012). This long lasting depolarized state of neurons is evoked by stimulation of Gq-coupled receptors (mGluRs, muscarine Ach, 5-HT receptors) (Zhang et al., 2013) that results in the activation of the IP3 pathway and release of Ca^++^ ions from the endoplasmic reticulum (Fowler et al., 2007). Elevation of the intracellular Ca^++^ concentration activates the calcium-activated non selective cation current (iCAN) (Haj-Dahmane and Andrade, 1998), possibly mediated by the transient receptors potential channels (TRPCs) of both the prefrontal and entorhinal neurons (Fowler et al., 2007; Zhang et al., 2011). In the PFC microcircuit model used here, persistent firing was induced in the absence of CAN current but required enhanced levels of NMDA receptor activation. Activating the CAN current allowed for persistent activity generation at lower NMDA-to-AMPA ratios, a similar finding observed in large-scale simplified networks (Tegnér et al., 2002). In addition, increasing the dADP values (within the normal physiological range) increased the firing frequency of persistent activity in agreement with earlier findings (Egorov et al., 2002). In sum, our results show that the dADP mechanism regulates the expression and properties of the persistent state in the microcircuit model.

The role of *I*_*h*_ in persistent activity in the microcircuit model is location-dependent: its reduction in the dendrites—but not the soma—lowers the induction probability. These findings are in agreement with some but not all experimental studies in the PFC, where the contribution of *I*_*h*_ to persistent firing remains unclear. Specifically, data from the Arnsten laboratory has shown that in layers I–III, an increase in dendritic *I*_*h*_ following α2A activation, decreases the contribution of a specific synaptic input to persistent firing, thus eliminating noise or distractors, while inhibiting *I*_*h*_ seems to promote persistent firing (Wang et al., 2007). On the other hand, *I*_*h*_ has been shown to directly participate in persistent firing induction in a subset of neurons (Winograd et al., 2008) by promoting their excitable state. Our data are in-line with the latter since reduction of dendritic *I*_*h*_ (but not somatic) lowers the induction probability. The observed differences could reflect region or layer specific differences of *I*_*h*_ in synaptic integration. Further experimental and/or computational studies will be required to resolve or clarify the role of *I*_*h*_ in persistent activity states.

D-type potassium channels also had a major effect on persistent activity. Blockade of these channels either at the dendrites or the soma greatly enhanced the probability for persistent activity emergence. This result is primarily attributed to the long-lasting negative regulation of excitability exerted by *I*_*D*_ on pyramidal model neurons, which counteracts the currents that support persistent firing (Yang et al., 1996). Interestingly, this current is selectively suppressed under dopamine application in PFC slices, through activation of D1 receptors (Dong and White, 2003), supporting the predicted need for controlling its value in order to allow the emergence of persistent activity.

Reducing the *I*_sAHP_ enhances the induction probability, particularly when blocked at the soma, thus indicating that attenuation of synaptic transmission also has a gating effect on synaptic persistent activity initiation. This is in line with earlier work where dendritic SK channel activation decreases excitatory synaptic transmission (Faber, 2010b), dopamine-mediated reduction of the sAHP increases synaptic gain *in vitro* (Thurley et al., 2008) and blockade of SK channels improves working memory *in vivo* (Brennan et al., 2008).

Blockade of voltage gated calcium channels in our model reduced both the fast and slow AHP, in agreement with experimental data (Sun et al., 2003; Hagenston et al., 2008; Faber, 2010a). On the contrary, increasing calcium channel conductance at the soma, where the effect of IsAHP is more prominent, increased the sAHP and decreased the induction probability. Dendritic modulation of VGCCs on the other hand had a more complex and type-specific effect. This may be attributed to the fact that dendritic intracellular calcium modulates both hyperpolarization and depolarization in these neurons: increase in dendritic, as opposed to somatic, calcium concentration is correlated with small amplitude of somatic hyperpolarization or even failure to activate SK channels (Hagenston et al., 2008). In addition, dendritic voltage and calcium depolarizing plateaus at the basal dendrites of layer V prefrontal pyramidal neurons are due to the activation of NMDA receptors and in great part insensitive to blockade of voltage gated calcium channels (Milojkovic et al., 2007). In accordance, our results show that dendritic blockade of N calcium channels does not affect persistent activity initiation and of L type only slightly reduces excitability, similarly to the IsAHP effect.

On the other hand, enhancement of the dendritic conductance of R type calcium channels facilitated persistent activity. R type calcium channels are known to promote bursting in hippocampal neurons (Metz et al., 2005) and possibly PFC pyramidal neurons (Sidiropoulou and Poirazi, 2012). Due to the absence of a specific blocker of these channels, their study is greatly hampered. In our study, we have uncovered a novel role of R-type calcium channels, and particularly dendritic R-type channels that of facilitating persistent activity that can possibly be examined experimentally.

In summary, we predict an important role for several diverse intrinsic mechanisms in both gating and modulating the probability of persistent activity emergence, which in most cases is at least indirectly supported by experimental findings. Specifically, our study reinforces the significant role of *I*_*h*_ in persistent firing, which should be examined further experimentally, while for the first time the D-type K current, the *I*_sAHP_ and the R-type calcium current are linked at the cellular level with modulating persistent activity emergence.

### What is next?

Several extensions to the basic idea deserve further consideration. One such idea is to thoroughly investigate the effect of network size effect on persistent activity emergence. Earlier studies have shown that persistent activity emerges either within large scale networks (Compte et al., 2000; Compte, 2006) or very small networks with just two cells and with unrealistically long synaptic delays (Gutkin et al., 2001). We showed in this study that when a basic microcircuit consisting of 7 pyramidal neurons and 2 inhibitory interneurons is activated by 90 excitatory synapses impinging on their apical dendrites of pyramidal neurons (with 10 synchronous events at 20 Hz) and the ratio of iNMDA/iAMPA is 2.3, then persistent activity is induced with a probability of 86%. What will happen to the persistent activity if the number of pyramidal neurons in the network is reduced or increased? Will the probability of induction increase or decrease? These are some of the questions that can be investigated in future studies.

It was beyond the scope of this work to incorporate and investigate the various phenomena involved in synaptic plasticity, such as the role of presynaptic GABA_B_ receptors, activity–dependent synaptic plasticity, or short-term plasticity. Nevertheless, we assume that implementation of physiological procedures that result in synaptic modifications may unravel other aspects of persistent activity features, such as its temporal irregularity. Moreover, the pyramidal models used here were chosen to be morphologically simplified, in order to dissect the role of intrinsic and synaptic currents from the contribution of a detailed dendritic morphology. Future investigations could address the role of synaptic plasticity in the induction of persistent activity in a microcircuit of pyramidal and inhibitory cells with detailed dendritic morphologies.

## Conclusion

In summary, computational studies investigating neuronal circuit dynamics have become more elaborate over the years (Sarid et al., 2007; Haeusler et al., 2009). The incorporation of anatomical, biophysical and connectivity constrains in these models will facilitate the search for the functional relevance of cortical microcircuits with respect to cognition. Our work predicts a new function for small-sized PFC microcircuits: to serve as the anatomical substrate for the expression and tunable modulation of persistent activity. Furthermore, our modeling work has revealed the role for several diverse ionic mechanisms, specifically the H-current, the D-type K current, the I_sAHP_ and the R-type calcium current, in persistent activity generation. The modulation of these reciprocal microcircuits during learning may serve as a mechanism for generating different subpopulations in the PFC that integrate inputs semi-independently, by using different persistent activity induction thresholds and generating firing patterns with different temporal dynamics.

### Conflict of interest statement

The authors declare that the research was conducted in the absence of any commercial or financial relationships that could be construed as a potential conflict of interest.
