# Supplementary material for: Induction and modulation of persistent activity in a layer V PFC microcircuit model
Source: Front Neural Circuits. 2013 Oct 9;7:161. doi: 10.3389/fncir.2013.00161 (PMC3793128; doi:10.3389/fncir.2013.00161)
Supplement: Supplementary file 1 [file Presentation1.PDF]

Supporting Online Material

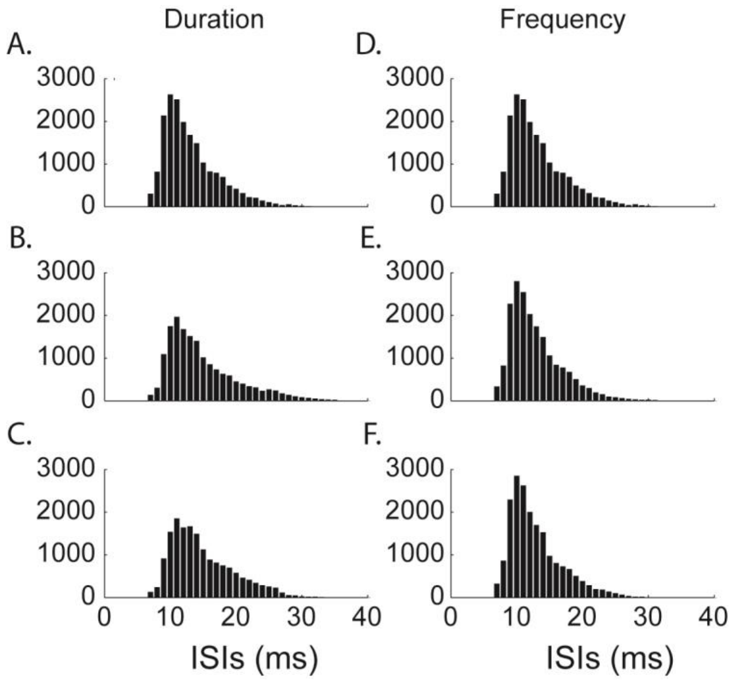

Supplemental Figure 1. Histograms of single-neuron ISIs showing the effect of stimulus duration and frequency.

For all iNMDA-to-iAMPA ratio=2.3, $iGABA_B$-to-$iGABA_A$ ratio=0.4, dADP deactivated. For A, B, C, stimulus frequency was 20Hz. For D, E, F, stimulus duration was 500ms. **A**. ISIs histograms for stimulus duration 500ms. **B.** ISIs histograms for stimulus duration 750ms. **C.** ISIs histograms for stimulus duration 1000ms. **D.** ISIs histograms for stimulus duration 20Hz **E.** ISIs histograms for stimulus duration 50Hz **F**.ISIs histograms for stimulus duration 100Hz.

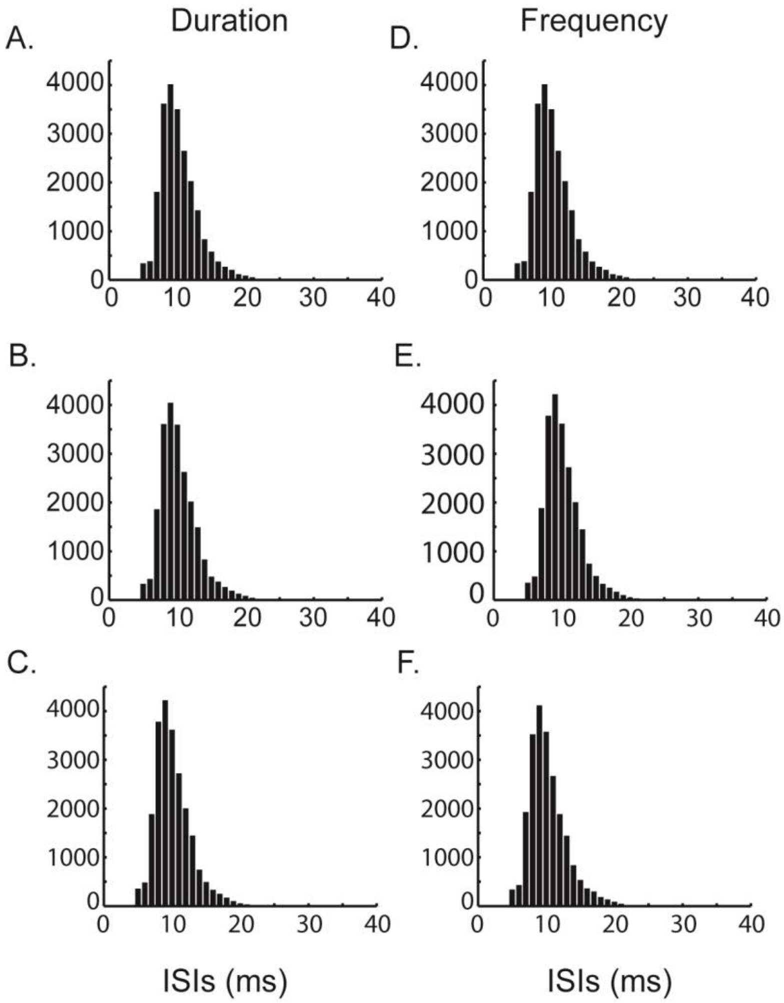

Supplemental Figure 2. Histograms of single-neuron ISIs showing the effect of stimulus duration and frequency, when dADP is activated.

For all iNMDA-to-iAMPA ratio=2.3, $iGABA_B$-to-$iGABA_A$ ratio=0.4, dADP 2mV. For A, B, C, stimulus frequency was 20Hz. For D, E, F, stimulus duration was 500ms. **A**. ISIs histograms for stimulus duration 500ms. **B.** ISIs histograms for stimulus duration 750ms. **C.** ISIs histograms for stimulus duration 1000ms. **D.** ISIs histograms for stimulus duration 20Hz **E.** ISIs histograms for stimulus duration 50Hz **F**.ISIs histograms for stimulus duration 100Hz.

Mathematical formalism

Pyramidal cell

The somatic (s), axonic (a), basal (bd) and apical (ad) dendritic compartments obey the following current balance equations:

$$C_m \frac{dV_s}{dt} = I_l + I_{Naf} + I_{NaP} + I_{Kdr} + I_D + I_A + I_{sAHP} + I_{fAHP} + I_{CaL} + I_{CaN} + I_{CaR} + I_{CaT} + I_h + I_{dADP} + I_{syn} \quad (1)$$

$$C_m \frac{dV_{bd}}{dt} = I_l + I_{Naf} + I_{NaP} + I_{Kdr} + I_D + I_A + I_{CaN} + I_h + I_{dADP} + I_{syn} \quad (2)$$

$$C_m \frac{dV_{ad}}{dt} = I_l + I_{Naf} + I_{NaP} + I_{Kdr} + I_D + I_A + I_{sAHP} + I_{fAHP} + I_{CaL} + I_{CaN} + I_{CaR} + I_{CaT} + I_h + I_{dADP} + I_{syn} \quad (3)$$

$$C_m \frac{dV_a}{dt} = I_l + I_{Naf} + I_{Kdr} \quad (4)$$

where I_l is the leak current, I_{Naf} is the fast sodium current, I_{Nap} is the persistent sodium current, I_{Kdr} is the delayed rectifier K^+ current, I_D and I_A are the D- and A-type K^+ currents, respectively, I_{sAHP} and I_{fAHP} are the slow and fast after-hyperpolarizing (AHP) currents, I_{caL}, $I_{caN,}$ I_{caT} and I_{caR} are the L-, N-, T- and R-type calcium currents, I_h is the h-current, I_{dADP} is the CAN current.

The leak current

$$I_l = g_l * (V - E_l) \quad (5)$$

where for pyramidal neurons E_l = -65 mV.

The fast sodium channel (Durstewitz & Gabriel, 2007)

$$I_{Naf} = g_{Na(fast)} * (V - E_{rev}) \quad (6)$$

$$g_{Na(fast)} = g * m^3 * h$$

$$\frac{dm}{dt} = \frac{m_\infty - m}{t_m}$$

$$m_\infty = \frac{ma}{ma + mb}$$

$$ma = \frac{-0.2816 * (v + 28)}{-1+e^{\frac{-v+28}{9.3}}}$$

$$mb = \frac{0.2464 * (v + 1)}{-1+e^{\frac{v+1}{6}}}$$

$$t_m = \frac{1}{ma + mb}$$

$$\frac{dh}{dt} = \frac{h_\infty - h}{t_h}$$

$$h_\infty = \frac{ha}{ha + hb}$$

$$ha = \frac{0.0.98}{e^{\frac{v+23.1}{20}}}$$

$$hb = \frac{1.4}{1+e^{\frac{-(v+25.1)}{10}}}$$

$$t_h = \frac{1}{ha + hb}$$

where E_{rev}=0 mV.

The persistent sodium channel (Durstewitz & Gabriel, 2007)

$I_{NaP} = g_{NaP} * (V - E_{rev})$ (7)

$$g_{NaP} = g * m * h$$

$$ma = \frac{-0.2816 * (v + 12)}{-1 + e^{\frac{-v+12}{9.3}}}$$

$$mb = \frac{0.2464 * (v - 15)}{-1 + e^{\frac{v-15}{6}}}$$

$$ha = 2.8e^{-5} * e^{\frac{-(v+42.8477)}{4.0248}}$$

$$hb = \frac{0.02}{1 + e^{\frac{-(v-413.9284)}{148.2589}}}$$

where E_{rev}= 0 mV and dm/dt, m_∞, t_m, dh/dt, h_∞, t_h as for fast sodium current.

.

The delayed rectifier potassium current (Durstewitz and Gabriel, 2007)

$I_{Kdr} = g_{Kdr} * (V - E_{rev})$ (8)

$$g_{Kdr} = gmax * n^4$$

$$\frac{dn}{dt} = \frac{n_\infty - n}{t}$$

$$n_\infty = \frac{na}{na + nb}$$

$$t = \frac{1}{na + nb}$$

$$na = \frac{-0.0.18*(v-13)}{-1 + e^{\frac{-(v+13)}{25}}}$$

$$nb = \frac{0.00544 * (v - 23)}{-1 + e^{\frac{(v-23)}{12}}}$$

where E_{rev} = 0 mV.

The fast inactivating potassium current (Poirazi, Brannon, & Mel, 2003)

$I_A = gmax * n * l * (V - E_K)$ (9)

$$\frac{dn}{dt} = \frac{n_\infty - n}{tn}$$

$$\frac{dl}{dt} = \frac{l_\infty - l}{tl}$$

$$n_\infty = \frac{1}{1+\alpha_n}$$

$$l_\infty = \frac{1}{1+\alpha_l}$$

$$tn = \frac{betn}{qt * 0.1 * (1+\alpha_n)}$$

$$betn = e^{\frac{0.001*\left(-1.8-\frac{1}{1+e^{\frac{(v+40)}{5}}}\right)*0.39*(v+1)*9.648e4}{8.315*(273.16+celsius)}}$$

$$q_t = 5^{\frac{celsius-24}{10}}$$

$$a_n = e^{\frac{0.001*\left(-1.8-\frac{1}{1+e^{\frac{(v+40)}{5}}}\right)*(v+1)*9.648e4}{8.315*(273.16+celsius)}}$$

$$t_l = \frac{0.26}{v + 50}$$

$$a_l = e^{\frac{0.001*3*(v+1)*9.648e4}{8.315*(273.16+celsius)}}$$

L-type calcium current (Poirazi et al., 2003)

$$I_{Ca(L)} = g * (m^2 * h^2 + s^2 * 8) * (V - E_{Ca}) \quad (10)$$

$$\frac{dm}{dt} = \frac{m_\infty - m}{t_m}$$

$$m_\infty = \frac{1}{1+a}$$

$$a = e^{\frac{0.001*-4.6*(v+1)*9.648e4}{8.315*(273.16+celsius)}}$$

$$\frac{ds}{dt} = \frac{s_\infty - s}{t_s}$$

$$s_\infty = \frac{alpha}{1+alpha}$$

$$alpha = \left(\frac{[Ca^{+2}]_i}{b}\right)^2$$

$$t_s = 180 + \frac{1}{[Ca^{+2}]i + 0.01}$$

$$h = \frac{0.025}{0.025 + [Ca^{+2}]_i}$$

where t_m=1.5 (ms).

N-type Calcium current (Poirazi et al., 2003)

$$I_{Ca(N)} = g * m^2 * h^2 * h2 * (V - E_{Ca}) \quad (11)$$

$$\frac{dm}{dt} = \frac{m_\infty - m}{t_m}$$

$$m_\infty = \frac{1}{1+a}$$

$$a = e^{\frac{0.001*-3.4*(v+21)*9.648e4}{8.315*(273.16+celsius)}}$$

$$\frac{dh}{dt} = \frac{h_\infty - h}{t_h}$$

$$h_\infty = \frac{1}{1+b}$$

$$b = e^{\frac{0.001*2*(v+40)*9.648e4}{8.315*(273.16+celsius)}}$$

$$h2 = \frac{0.025}{0.025 + [Ca^{+2}]_i}$$

where t_m = 1.5 ms and t_h = 75 ms and dm/dt, m_∞ as for L-type calcium current.

R-type calcium current (Poirazi et al., 2003)

$$I_{Ca(R)} = g * m^3 * h(V - E_{Ca}) \quad (12)$$

$$E_{CaR} = 1\frac{e^3 * R * (celsius + 273.15)}{2 * Faraday} * log\left(\frac{[Ca^{+2}]_o}{[Ca^{+2}]_i}\right)$$

$$m_\infty = \frac{1}{1+e^{\frac{-(v+43.5)}{3}}}$$

$$h_\infty = \frac{1}{1+e^{v+50}}$$

where t_m = 70 ms and t_h = 20 ms and dm/dt, dh/dt as for N-type calcium current.

Low threshold activated calcium current (Poirazi et al., 2003)

$$I_{Ca(R)} = g * m^2 * h * (V - E_{Ca}) \quad (13)$$

$$m_\infty = \frac{1}{1+a}$$

$$a = e^{\frac{0.001*-3*(v+36)*9.648e4}{8.315*(273.16+celsius)}}$$

$$h_\infty = \frac{1}{1+b}$$

$$b = e^{\frac{0.001*5.2*(v+68)*9.648e4}{8.315*(273.16+celsius)}}$$

where t_m = 1.5 ms, t_h = 10 ms and dm/dt, dh/dt as for N-type calcium current.

The fast calcium dependent potassium current (IfAHP) (Shao et al., 1999)

$$I_{fAHP} = g * O * (V - E_K) \quad (14)$$

The channel has three states: active (O), non active (C) and inactivated (I).

The transition from C to O is calcium dependent. The transition from O to I is voltage dependent and is responsible for the presence of the fast afterhyperpolarization potential. The transition from I to C is voltage dependent and slow as well.

$C \leftrightarrow O\ (K_3,\ K_4)$

$O \leftrightarrow I\ (K_1,\ 0)$

$I \leftrightarrow C\ (K_2,\ 0)$

$C + O + I = 1$

where K_1, K_2, K_4 are described by function a_2 and K_3 by function a_1 and correspond to the transition rates between open (O), close (C) and inactivated (I) states:

$$a_1 = \frac{1}{tmin + \frac{1}{\frac{1}{(tmax - tmin)} + e^{\frac{(v - vhalf)}{K}}}} * 1e8 * ([Ca^{+2}]_i)^3$$

$$a_2 = \frac{1}{tmin + \frac{e^{-(v - vhalf)}}{K}}$$

Where

	(ms)	(ms)	(mV)	K (mV)
K1	0.1	-	-10	1.0
K2	0.1	-	-120	-10.0
K3	0.001	1.0	-20	7.0
K4	0.2	-	-44	-5.0

and $[Ca^{+2}]_i$ is the internal calcium concentration (mM).

The slow calcium dependent potassium channel I_{sAHP} (Poirazi et al., 2003)

$$I_{sAHP} = g * m^2 * (V - E_{Ca}) \qquad (15)$$

$$m_\infty = \frac{a}{a+1}$$

$$a = \frac{[Ca^{+2}]_i}{0.008}$$

$$t_m = \frac{0.008}{[Ca^{+2}]_i + 0.008}$$

where $[Ca^{+2}]_i$ is the internal calcium concentration (mM) and dm/dt as for N-type calcium current.

Hyperpolarization Activated Current I_h (Poirazi et al., 2003)

$$I_h = g * n * (V - E_h) \qquad (16)$$

$$\frac{dn}{dt} = \frac{n_\infty - n}{t_n}$$

$$n_\infty = 1 - \frac{1}{1+e^{\frac{(-90-v)}{10}}}$$

$$t_n = 2 * \left(\frac{1}{e^{\frac{(v+145)}{-17.5}} + e^{\frac{v+16.8}{16.5}}} + 10 \right)$$

else when v>-10 t_n=1 (ms). The reversal potential, E_h = -10 mV.

Slow inactivating potassium current (I_D) (Durstewitz & Gabriel, 2007)

$$I_D = g * a * b * (V - E_K) \tag{17}$$

$$\frac{da}{dt} = \frac{a_\infty - a}{t_a}$$

$$\frac{db}{dt} = \frac{b_\infty - b}{t_b}$$

$$a_\infty = \frac{1}{1+e^{\frac{(v+34)}{6.5}}}$$

$$b_\infty = \frac{1}{1+e^{\frac{(v+65)}{6.6}}}$$

$$t_b = 200 + \frac{3200}{1+e^{\frac{-(v+63.6)}{4}}}$$

where t_a=10 ms.

The dADP (CAN) mechanism (Sidiropoulou & Poirazi, 2012)

$$I_{n(CAN)} = g * m^2 * (V - E_n) \tag{18}$$

$$I_{Na} = 0.7 * I_{n(CAN)}$$

$$m_\infty = \frac{alpha}{alpha + beta}$$

$$t_m = \frac{1}{\frac{alpha + beta}{tadj}}$$

$$alpha = beta * \left(\frac{[Ca^{+2}]_i}{cac}\right)^2$$

$$tadj = 3^{\frac{celsius-22}{10}}$$

where beta = 0.0001 1/ms and cac = 0.0004 mM and dm/dt as for N-type calcium current.

Inhibitory interneuron

The somatic (s) and axonic (a) compartments obey the following current balance equations:

$$C_m \frac{dV_s}{dt} = I_l + I_{Naf} + I_{Kdr} + I_D + I_{syn} \quad (19)$$

$$C_m \frac{dV_a}{dt} = I_l + I_{Naf} + I_{Kdr} \quad (20)$$

where I_l is the leak current, I_{Naf} is the fast sodium current, I_{Kdr} is the delayed rectifier K^+ current and I_D is the D-type K^+ current.

The leak current

$$I_l = g_l * (V - E_l) \quad (21)$$

where E_l = -70mV.

The fast sodium channel (Durstewitz & Gabriel, 2007)

$$I_{Naf} = g_{Na(fast)} * (V - E_{rev}) \quad (22)$$

$$g_{Na(fast)} = g * m^3 * h$$

$$\frac{dm}{dt} = \frac{m_\infty - m}{t_m}$$

$$m_\infty = \frac{ma}{ma + mb}$$

$$ma = \frac{-0.2816 * (v + 28)}{-1 + e^{\frac{-v+28}{9.3}}}$$

$$mb = \frac{0.2464 * (v + 1)}{-1 + e^{\frac{v+1}{6}}}$$

$$t_m = \frac{1}{ma + mb}$$

$$\frac{dh}{dt} = \frac{h_\infty - h}{t_h}$$

$$h_\infty = \frac{ha}{ha + hb}$$

$$ha = \frac{0.0.98}{e^{\frac{v+23.1}{20}}}$$

$$hb = \frac{1.4}{1 + e^{\frac{-(v+25.1)}{10}}}$$

$$t_h = \frac{1}{ha + hb}$$

where E_{rev}=0 mV.

The delayed rectifier potassium current (Durstewitz and Gabriel, 2007)

$I_{Kdr} = g_{Kdr} * (V - E_{rev})$ (23)

$$g_{Kdr} = gmax * n^4$$

$$\frac{dn}{dt} = \frac{n_\infty - n}{t}$$

$$n_\infty = \frac{na}{na + nb}$$

$$t = \frac{1}{na + nb}$$

$$na = \frac{-0.0.18 * (v - 13)}{-1 + e^{\frac{-(v+13)}{25}}}$$

$$nb = \frac{0.00544 * (v - 23)}{-1 + e^{\frac{(v-23)}{12}}}$$

where E_{rev}= 0 mV.

Slow inactivating potassium current (I_D) (Durstewitz & Gabriel, 2007)

$I_D = g * a * b * (V - E_K)$ (24)

$$\frac{da}{dt} = \frac{a_\infty - a}{t_a}$$

$$\frac{db}{dt} = \frac{b_\infty - b}{t_b}$$

$$a_\infty = \frac{1}{1 + e^{\frac{(v+34)}{6.5}}}$$

$$b_\infty = \frac{1}{1 + e^{\frac{(v+65)}{6.6}}}$$

$$t_b = 200 + \frac{3200}{1+e^{\frac{-(v+63.6)}{4}}}$$

where t_a = 10 ms.

Synaptic currents

NMDA receptor (pyramidal neurons) (Polsky, Mel, & Schiller, 2009)

$I_{nmda} = g \times (V - E_{rev})$ (25)

$$i[Ca^{+2} = \frac{inmda}{10}$$

$$g = \frac{A - B}{1 + n * e^{-gamma*v}}$$

$$\frac{dA}{dt} = \frac{-A}{tau1}$$

$$\frac{dB}{dt} = \frac{-B}{tau2}$$

where E_{rev} = 0 mV, tau1 = 90 ms, tau2 = 5 ms, gamma = 0.08 mV^{-1}, n = 0.25 mM^{-1}.
Note that the conductance of the NMDA is multiplied by the term $1/(1 + n * e^{-gamma*v})$ that give a sigmoidal voltage-dependence (including the Mg^{+2} blocking) (Polsky et al., 2009).

NMDA receptor (interneurons) (Destexhe, Mainen, & Sejnowski, 1994)

$I_{nmda} = g \times (V - E_{rev})$ (26)

$$ica = \frac{7 * Inmda}{10}$$

$$g = (R_{on} + R_{off}) * B$$

$$\frac{dR_{on}}{dt} = \frac{R_{inf} - R_{on}}{R\tau}$$

$$\frac{dR_{off}}{dt} = -\beta * R_{off}$$

$$R_{inf} = \frac{Cmax * \alpha}{Cmax * \alpha + \beta}$$

$$R\tau = \frac{1}{\alpha * Cmax + \beta}$$

$$B = \frac{1}{1 + \frac{e^{-0.072v} * [Mg^{+2}]}{3.57}}$$

where Mg^{+2} = 1 mM, Cmax = 1 mM, α = 4 $ms^{-1}mM^{-1}$, β = 0.015 ms^{-1}, and E_{rev}= 0 mV.

AMPA receptor (Destexhe et al., 1994)

$I_{AMPA} = g \times (V - E_{rev})$ (27)

$$g = R_{on} + R_{off}$$

$$\frac{dR_{on}}{dt} = \frac{R_{inf} - R_{on}}{R\tau}$$

$$\frac{dR_{off}}{dt} = -\beta * +R_{off}$$

$$R_{inf} = \frac{Cmax * \alpha}{Cmax * \alpha + \beta}$$

$$R\tau = \frac{1}{\alpha * Cmax + \beta}$$

where for pyramidal neurons E_{rev}=0 mV , α = 10 ms^{-1}, β = 0.15 ms^{-1}, C_{max}= 1 mM. For inhibitory interneurons E_{rev}= 0 mV, α = 10 ms^{-1}, β = 0.18 ms^{-1}, and C_{max}=1 mM.

GABA$_A$ receptor (Destexhe et al., 1994)

$$I_{GABAA} = g \times (V - E_{rev}) \quad (28)$$

$$g = R_{on} + R_{off}$$

$$\frac{dR_{on}}{dt} = \frac{R_{inf} - R_{on}}{R\tau}$$

$$\frac{dR_{off}}{dt} = -\beta * R_{off}$$

$$R_{inf} = \frac{Cmax * \alpha}{Cmax * \alpha + \beta}$$

$$R\tau = \frac{1}{\alpha * Cmax + \beta}$$

where for pyramidal neurons E_{rev} = -80 mV, α = 5 ms^{-1}, β = 0.18 ms^{-1}, and C_{max}= 1 mM. For inhibitory interneurons E_{rev} = -80 mV, α = 5 ms^{-1}, β = 0.2 ms^{-1}, C_{max}= 1 mM.

GABA$_B$ receptor (Pissadaki, Sidiropoulou, Reczko, & Poirazi, 2010)

$$I_{GABAB} = g \times (V - E_{rev}) \quad (29)$$

$$g = R_{on} + R_{off}$$

$$\frac{dR_{on}}{dt} = \frac{R_{inf} - R_{on}}{R\tau}$$

$$\frac{dR_{off}}{dt} = -\beta * R_{off}$$

$$R_{inf} = \frac{Cmax * \alpha}{Cmax * \alpha + \beta}$$

$$R\tau = \frac{1}{\alpha * Cmax + \beta}$$

where E_{rev} = -80 mV, α = 0.001 ms^{-1}, β = 0.0047 ms^{-1}, and C_{max}= 10 mM.

References

Destexhe, A., Mainen, Z. F., & Sejnowski, T. J. (1994). Synthesis of models for excitable membranes, synaptic transmission and neuromodulation using a common kinetic formalism. Journal of computational neuroscience, 1(3), 195–230.

Durstewitz, D., & Gabriel, T. (2007). Dynamical basis of irregular spiking in NMDA-driven prefrontal cortex neurons. Cerebral cortex, 17(4), 894–908. doi:10.1093/cercor/bhk044

Pissadaki, E. K., Sidiropoulou, K., Reczko, M., & Poirazi, P. (2010). Encoding of spatio-temporal input characteristics by a CA1 pyramidal neuron model. (L. J. Graham, Ed.)PLoS computational biology, 6(12), e1001038. doi:10.1371/journal.pcbi.1001038

Poirazi, P., Brannon, T., & Mel, B. W. (2003). Online Supplement : About the Model 1 Building the Model. Neuron, 37(2).

Polsky, A., Mel, B. W., & Schiller, J. (2009). Encoding and decoding bursts by NMDA spikes in basal dendrites of layer 5 pyramidal neurons. The Journal of Neuroscience, 29(38), 11891–903. doi:10.1523/JNEUROSCI.5250-08.2009

Shao, L. R., Halvorsrud, R., Borg-Graham, L., & Storm, J. F. (1999). The role of BK-type Ca2+-dependent K+ channels in spike broadening during repetitive firing in rat hippocampal pyramidal cells. The Journal of physiology, 521 Pt 1, 135–46.

Sidiropoulou, K., & Poirazi, P. (2012). Predictive Features of Persistent Activity Emergence in Regular Spiking and Intrinsic Bursting Model Neurons. (A. Morrison, Ed.)PLoS Computational Biology, 8(4), e1002489. doi:10.1371/journal.pcbi.1002489
